# Safety and quality outcomes of same-day discharge for gender-affirming mastectomy during the pandemic

**DOI:** 10.1038/s41598-025-28462-z

**Published:** 2025-11-29

**Authors:** Holly Christopher Lewis, Alexandria Mullikin, Lulla Kiwinda, Koumani Ntowe, Christian Bailey-Burke, William Tian, Carmen Kloer, Andrew Hollins, Kristen Rezak

**Affiliations:** 1 Department of Intensive Care and Resuscitation, Cleveland Clinic, Cleveland, OH USA; 2 Division of Plastic, Maxillofacial and Oral Surgery, Department of Surgery, Duke University Medical Center, Durham, NC USA

**Keywords:** Medical research, Outcomes research, Quality of life

## Abstract

The COVID-19 pandemic led to widespread postponement of elective procedures—including transplant, oncologic, and breast surgeries—prompting global expansion of ambulatory discharge pathways to preserve capacity and reduce exposure. However, the safety of this approach for transgender and gender-diverse (TGD) patients has not been well established. Gender-affirming mastectomy is a common procedure for TGD individuals, yet perioperative outcomes during the pandemic period remain poorly characterized. While several studies have examined ambulatory mastectomy in oncologic or reconstructive contexts, few have evaluated pandemic-era shifts in discharge practices or their implications for safety and patient experience in gender-affirming surgery. A retrospective review was conducted of 57 consecutive adults who underwent gender-affirming mastectomy at a single academic center from 2018 to 2021, spanning pre- and peri-pandemic periods. Primary outcomes included postoperative complications and unplanned health care utilization within 30 days (emergency department visit, reoperation, or patient-initiated call/message). Chart-documented misgendering (use of incorrect name or pronouns) was evaluated as a cultural complication. Forty-five patients (79%) were discharged the same day and 12 (21%) stayed overnight. Complications occurred in 2.2% of ambulatory vs. 8.3% of overnight cases, an absolute difference of 6% points. Although this difference did not achieve statistical significance (*p* = 0.38), the effect magnitude suggests a potential clinical signal that a larger, adequately powered study could better define. Unplanned emergency visits (8.9% vs. 16.7%, *p* = 0.60) and reoperations (0% vs. 8.3%, *p* = 0.22) showed similar directional patterns. Taken together, these trends indicate possible differences in postoperative trajectories that may be obscured by the limited power of the current analysis. Patient-initiated calls or messages occurred in 71.1% of ambulatory and 41.7% of overnight cases, suggesting possible differences in reassurance-seeking or access to remote communication. Misgendering appeared in 38.6% of charts overall and was more frequently documented among overnight patients (66.7% vs. 22.2%), an absolute difference of ≈ 45% points (*p* = 0.04). We conducted a retrospective cohort study examining perioperative outcomes during a period of restricted surgical volume associated with the COVID-19 pandemic. Within the inherent limitations of pandemic-era cohort size and inferential power, same-day discharge after gender-affirming mastectomy was implemented without clear evidence of increased short-term surgical complications or unplanned health care utilization. The trends observed in our patient population may nonetheless hold clinical relevance and warrant confirmation in larger, adequately powered studies. The higher frequency of documented misgendering among overnight patients underscores both the cultural and clinical dimensions of postoperative and psychological safety. Taken together, these findings suggest that ambulatory management is feasible and promising for appropriately selected transgender and gender-diverse patients, while emphasizing the need for multi-institutional research to better estimate effect sizes and to strengthen affirming communication practices across care settings.

## Introduction

 In the early days of the COVID-19 pandemic, elective surgeries at U.S. medical centers were canceled, postponed or rescheduled for the protection of patients and health care workers alike^1^. The American College of Surgeons and American Society of Plastic Surgeons advised triaging of surgical procedures to safeguard patients and health care workers^2,3^. Some health care delays, notably those related to gender-affirming health care (including mastectomy) were at least partially mitigated by telehealth expansion^4^.

Access to gender-affirming surgery is widely recognized as an essential component of well-being and psychosocial health for transgender and gender-diverse (TGD) individuals^5–11^. Conversely, interruptions or delays in this care have been associated with negative psychosocial and health outcomes, underscoring the importance of efficient and affirming surgical delivery models. Disruptions or delay in gender-affirming care has potentially negative consequences^12,13^. Therefore, there is need for efficient health care delivery frameworks that find ways to maximize patient safety while minimizing hospital staff burden.

Since the pandemic, health systems developed testing and risk management systems, returning academic centers to a pre-pandemic case-volume, while prioritizing patient and personnel safety. Within the authors’ institution, we observed changing practice patterns, such that oncologic and gender-affirming mastectomies moved from being an overnight-observation procedure to primarily an ambulatory surgery (same-day discharge). This transition paralleled national shifts toward ambulatory management intended to preserve inpatient capacity and minimize exposure risk, but its effects on outcomes in TGD surgery have not been well characterized. A concern of patients and health care workers is whether this correlates with increased risk of postoperative complications, e.g. hemorrhage necessitating unplanned return to the operating room. Others have advocated that an overnight stay can be beneficial for patients whose pain is not well-controlled with oral medications. Numerous studies have examined the safety implications of ambulatory breast surgery compared to inpatient/overnight breast surgery for oncologic purposes, finding that for select patients, it can be a safe practice^14,15^. Additionally, several authors have published on the safety of tissue expander-based breast reconstruction in the setting of ambulatory mastectomy^16,17^. However, these studies were performed in cisgender patient populations and primarily evaluated conventional surgical outcomes such as hematoma or infection, without examining patient experience or affirming care processes. As such, whether comparable findings apply to gender-affirming mastectomy remains uncertain.

In the United States, transgender health research often faces methodological challenges, including small sample sizes, incomplete gender identity data, and under-representation in large clinical databases. This persists despite recent evidence that nearly three million Americans, or approximately 1% of the population, identify as transgender or nonbinary, underscoring a major disconnect between population prevalence and data visibility^18^. These structural limitations, compounded by stigma and inconsistent institutional infrastructure, contribute to persistent data gaps in perioperative outcomes research. Traditional large-scale randomized controlled trials or high-powered population-level cohort studies, which form the foundation of conventional statistical inference, are often infeasible in this population due to under-ascertainment in health records and barriers to inclusive recruitment. As a result, many studies in transgender health rely on descriptive or exploratory approaches designed to identify trends and generate hypotheses rather than establish causal relationships.

An unanswered question is how ambulatory discharges may impact quality of care and patient experience, especially among patients who may have further questions or concerns that could otherwise be addressed by overnight clinicians in a postoperative observation unit. Accordingly, within the setting of increased reliance on ambulatory discharge pathways, we aimed to descriptively characterize unplanned health care utilization among TGD patients, focusing on the frequency of patient-initiated phone calls and secure electronic health record (EHR) messages within the early postoperative period.

Given the unique risk profiles related to TGD patients, we sought to focus our research efforts on this population. A singular study focusing on the transgender population has demonstrated the safety of same-day discharge for gender-affirming facial surgery^19^, but we identified no studies exploring ambulatory mastectomy and its impact on TGD patient outcomes and experiences. To our knowledge, this represents an unaddressed area within the gender-affirming surgery literature and an opportunity to contribute descriptive evidence toward improving perioperative care models.

TGD patients are a vulnerable population with unique health care needs, requiring attention to basic demographics such as name, pronouns, procedure and comorbidities. Prior work has shown that medicalized trauma, defined as distress or mistrust arising from stigmatizing or discriminatory experiences in health care settings, remains common in this community; studies indicate that nearly one-third of transgender patients delay necessary medical care due to fear of re-experiencing such treatment^16^. As our practice evolved, we learned that our transgender patients were sometimes experiencing misgendering events, at verbal handoffs in post-anesthesia care unit, or in written notes on the surgical floor.

While the root causes of misgendering and medical trauma are multifactorial and embedded in systemic and cultural structures, reducing the duration of inpatient exposure may lessen the number of clinical interactions in which such events could occur. However, earlier discharge cannot in itself prevent misgendering or address its underlying causes. Building on patient feedback and discussions with clinicians of transgender experience, we sought to explore whether shorter inpatient exposure correlated with fewer documented misgendering events. Assuming similar medical risks, we hypothesized that a patient might feel more psychological safety being discharged directly home with their support person or family member than when admitted for overnight observation.

Given the limited sample size available during pandemic-related restrictions, we anticipated that this analysis would be exploratory and hypothesis-generating rather than confirmatory. Accordingly, the study was designed to identify descriptive patterns in safety, health care utilization, and cultural complications (such as misgendering) between ambulatory and overnight groups, while acknowledging that the sample was underpowered to detect smaller effect sizes. Additionally, with the increased use of telemedicine since the COVID-19 pandemic alongside the return to in-person care, it is important to assess how telemedicine usage is evolving in a post-pandemic society. Within this broader context, we sought to examine how pandemic-era shifts toward ambulatory management intersected with gender-affirming care delivery and communication patterns in a TGD surgical cohort.

## Methods

### Study population

Using the DEDUCE platform, a data set was created comprising all patients at Duke Health who underwent unilateral or bilateral mastectomy, defined by current procedural terminology (CPT) codes 19,303, 19,304, 19,318, then selecting for those patients who underwent surgery for gender-affirming indications^20^. The time frame of cases was centered around the statewide stay-at-home order for the state of North Carolina announced by the governor on March 30, 2020. Data from the preceding 18-month time interval serves as the baseline pre-pandemic cohort, with the following 18-month time interval as the post-pandemic cohort. Data included surgical patients from a variety of surgical centers within our health system; all gender-affirming mastectomies were performed by the senior author. All patients were adults over the age of 18 who identified as transgender or gender diverse (TGD) and sought bilateral mastectomy for gender affirmation. This cohort represents the complete institutional experience of gender-affirming mastectomy during the peri-pandemic period and was analyzed descriptively to explore potential differences in outcomes between discharge groups, recognizing that inferential power is limited by sample size.

### Variables and outcomes

The following demographics were collected: age at the time of the surgery, BMI, sex assigned at birth, gender identity, racial identity, smoking status (active, former, or never smoker), history of diabetes, history of coagulopathy (defined as deep vein thrombosis or pulmonary embolism, Factor V Leiden, von Willebrand factor disease, or other coagulopathy), or use of an oral anticoagulant or antiplatelet drug. Ambulatory patients were defined as those who were discharged from the hospital on the same day as their surgery, while overnight patients were defined as those who spent at least one night in the hospital after surgery. Incision design (periareolar vs double incision) and the use of local nerve blocks were recorded.

Postoperative complications were defined as hematoma, seroma, wound dehiscence, surgical site infection, and deep venous thrombosis. Given the retrospective and observational design of this study, comparisons between groups were performed to explore potential clinical trends rather than to establish definitive statistical differences. Incidence of misgendering in TGD patients was considered a cultural complication^21^, with the ‘gold standard’ defined by the patient’s name and pronouns in the electronic health record^22^, as documented at the most-recent preoperative clinic note, and corroborated by the attending surgeon’s history and physical and operative report notes from the date of surgery. Notes that referred to a patient using non-affirmed name or inaccurate pronouns were defined as a deadnaming event. All such notes are visible to patients in their mobile devices and patient portals, and therefore constitute a potential means of conferring psychological harm^23^. Unrecorded or undocumented misgendering events could not be captured, and this limitation is explicitly acknowledged in interpretation of findings.

Unplanned health care utilization was defined as return to the emergency department, return to the operating room, or a call/message to the provider within 30 days. The primary outcomes measured were length of hospital stay in days, postoperative complications, and unplanned health care utilization. Quality-of-care metrics were defined as unplanned emergency department visits, patient-initiated messages or phone calls within 30 days, and unplanned returns to the operating room.

Racial identity was documented for all patients and is reported in the Results section. This study was conducted as part of a larger analysis that included both oncologic and gender-affirming patients who underwent mastectomy during the peri-pandemic period. Accordingly, all patients were evaluated for potential coagulopathic risk factors (e.g., Factor V Leiden, von Willebrand disease, or use of oral anticoagulant or antiplatelet therapy). Within the present cohort (gender-affirmation patients), none had a recorded history of inherited or acquired coagulopathy or were prescribed an oral anticoagulant or antiplatelet agent at the time of surgery; therefore, this variable was omitted from final comparative analyses. All but one patient (*N* = 56) received intraoperative local field or muscle blocks using liposomal bupivacaine administered by the operating surgeon, and one patient underwent an anesthesiology-performed regional block. Given the very low utilization of this modality, formal statistical comparison of analgesia techniques was not performed.

### Statistical analysis

Categorical variables were summarized as n (%). Continuous variables were summarized with mean ± SD. Associations with postoperative complications were explored descriptively using Fisher’s exact test for categorical variables and Mann–Whitney U or chi-square tests where appropriate. A *p* value ≤ 0.05 was used to define statistical significance; however, given limited power, all analyses were interpreted as exploratory and hypothesis-generating rather than confirmatory. Statistical software included Microsoft Excel 16.7 (Redmond, WA), REDCap (Nashville, TN), Duke Protected Analytics Computing Environment (PACE) (Durham, NC).

### Power and sample-size considerations

This retrospective cohort represents all gender-affirming mastectomy cases performed at our institution during a three-year peri-pandemic interval (2018–2021). The sample size therefore reflects the total number of eligible procedures performed in that period, during which elective surgical volume was sharply restricted by institutional and national COVID-19 policies. A power analysis was conducted using G*Power version 3.1 (Heinrich Heine University, Düsseldorf, Germany). This analysis confirmed that the study was underpowered to detect small or moderate population effects, and only large absolute differences (≥ 15–20%) in outcomes would likely reach significance at α = 0.05. For example, detecting an absolute 10-percentage-point difference in 30-day complication rates (10% vs. 20%) with 80% power would require approximately 195 patients per group; with the observed 45:12 distribution, estimated power to detect 10–15% differences was < 20%. Accordingly, the analyses presented herein are descriptive and exploratory in nature, intended to contextualize institutional experience rather than to infer population-level effects.

## Results

### Study population

A total of 57 patients from a single institution underwent gender-affirmingmastectomy during the study period. Overall, 35% identified as non-binary or gender-fluid, and 65% identified as male. Of these, 45 (78.9%) were discharged the sameday (ambulatory group), and 12 (21.1%) remained for overnight observation. Allpatients were older than 18 years at the time of surgery, with a mean age of 26 years. Mean BMI was 26.13 for ambulatory patients and 25.67 for overnight patients. Onlythree individuals were active smokers, all within the ambulatory group. Racial identityis summarized in Table 1. One patient was diabetic and had an overnight stay (Table 2). No patient had a recorded history of inherited or acquired coagulopathy (e.g.,Factor V Leiden or von Willebrand disease) or use of oral anticoagulant or antiplatelettherapy; this variable was therefore omitted from comparative analysis.

Nearly all patients (N = 56) received intra-operative local field or muscle blocks with liposomal bupivacaine administered by the operating surgeon, and one patient received an anesthesiology-performed regional block. Given that utilization of regional anesthesia was rare, no subgroup comparisons were pursued. All patients were discharged with standardized prescriptions for oral analgesics, antiemetics, and stool softeners determined by the attending surgeon. Prescription refills after discharge could not be retrieved in this dataset and were therefore excluded from structured analysis.

### Postoperative complications

Across the cohort, postoperative complications were infrequent, occurring in one patient per group. One ambulatory patient (2.2%) experienced wound dehiscence, and one overnight patient (8.3%) experienced a hematoma requiring reoperation. No cases of seroma, surgical-site infection, or deep venous thrombosis were identified within 30 days (Table 3). Although absolute rates of complication appeared slightly higher in the overnight group (8.3% vs 2.2%), this difference did not reach statistical significance (*p* = 0.38) and should be interpreted descriptively given limited power.

### Health care utilization

Unplanned emergency-department visits occurred in 8.9% of ambulatory patients and 16.7% of overnight patients, while reoperations occurred in 0% and 8.3%,respectively. Although these comparisons did not reach statistical significance(*p* = 0.60 and *p* = 0.21), the absolute differences—ranging from 7 to 8 percentage points—could reflect clinically meaningful trends that the present study was underpowered to detect. Patient-initiated telephone calls or portal messages were documented for 71.1% of ambulatory patients and 41.7% of overnight patients, a 29-point absolute difference that, while not statistically significant (*p *= 0.42), suggests potential variation in postoperative reassurance-seeking or access to remote communication rather than true differences in complication burden. Given small and uneven group sizes, these findings should be interpreted as preliminary indicators of possible effect direction and magnitude rather than as evidence of equivalence or difference.

### Incision design and nipple reconstruction

Demographic and technical variables were further examined for potential association with postoperative outcomes. Incision designs included periareolar, double-incision, wise-pattern, and buttonhole techniques. Among non-binary patients, 20% underwent mastectomy without nipple-areola complex reconstruction. The double-incision approach was most common (n = 37, 65.9%), followed by periareolar (n = 15,26.3%). Two of fifteen periareolar cases (13.3%) and three of thirty-seven double-incision cases (8.1%) were associated with complications; none occurred in the wise-pattern or buttonhole subgroups. No statistically significant differences wereobserved among incision types (Table 4), though the sample size limits detection of modest effect sizes.

### Nicotine use

Within the cohort, only three patients reported active smoking. One of these three(33%) experienced a postoperative complication, aligning with established evidence linking nicotine exposure to impaired wound healing, vasoconstriction, and higher complication rates in surgical patients^24,25^. Rates of nicotine and tobacco use among transgender and gender-diverse (TGD) individuals have been shown in multiple population-based surveys to exceed those of cisgender peers^26,27.^ Preoperative nicotine cessation requirements and urine cotinine screening are common practices in gender-affirming surgical programs^28^, and may inadvertently encourage under-reporting of tobacco use by patients concerned about delays or denials of care. This pattern represents a potential source of bias in retrospective datasets derived from self-reported substance use histories and may partially obscure the true relationship between nicotine exposure and postoperative risk in TGD surgical cohorts.

### Cultural complications (misgendering)

Across the entire cohort, 22 patients (38.6%) experienced at least one misgendering event, defined as deadnaming or incorrect pronoun usage in written documentation within the EHR. Misgendering occurred in 10 of 45 ambulatory cases (22.2%) and in8 of 12 overnight cases (66.7%), representing a statistically significant difference (*p* = 0.04) that, while modest in magnitude, may reflect greater exposure to documentation errors among patients remaining in inpatient settings. Operative and clinic notes authored by the attending surgeon contained no misgendering events, and all corresponded with affirmed name and pronouns. Undocumented instances of misgendering could not be assessed, and the observed frequencies likely underestimate the true prevalence of such events. Taken together, these findings indicate that both clinical and cultural complications were relatively uncommon in this institutional cohort, with observed differences best interpreted as descriptive trends warranting further evaluation in larger, adequately powered studies.

## Discussion

This study represents a contemporary cohort examining ambulatory mastectomy specifically for TGD patients. We observed postoperative complications in both ambulatory and overnight cohorts following gender-affirming mastectomy, with low absolute event counts overall (one case in each group). The observed complication rates were 2.2% in the ambulatory group and 8.3% in the overnight group. Although this absolute difference did not reach statistical significance in our low-powered sample study, differences of this magnitude may not be sufficient to reject a null hypothesis – that is, they could be clinically meaningful at a population level, given large study design. Hematoma requiring reoperation occurred in the overnight group and not in the ambulatory group, whereas wound dehiscence occurred in the ambulatory group and not in the overnight group. No cases of seroma, surgical-site infection, or deep venous thrombosis were identified within 30 days (Table 3). Rather than concluding that outcomes are equivalent between groups, these data are best interpreted as descriptive estimates that inform effect size and direction for future adequately powered studies.

Our findings align broadly with prior studies evaluating ambulatory breast surgery in cisgender patients, where same-day discharge did not increase reoperation or readmission risk^14,15^. In oncologic cohorts, same-day mastectomy has been associated with reduced costs and decreased inpatient opioid use^14^. While our absolute event frequencies suggest a similar safety profile, the present analysis cannot exclude smaller yet meaningful population effects due to low statistical power. Importantly, prior studies were performed exclusively in cisgender populations, underscoring the need to evaluate whether comparable outcomes apply to TGD patients receiving gender-affirming surgical care. Similarly, recent analyses of Enhanced Recovery After Surgery (ERAS) pathways for chest reconstruction have demonstrated reduced length of stay without increased complications^29^. Our study contributes complementary evidence from an affirming-care cohort, suggesting that ambulatory or short-stay pathways may be feasible and safe when applied in appropriately-selected cases, but confirmation may require larger, multicenter data.

Unplanned emergency-department visits occurred in 8.9% of ambulatory patients and 16.7% of overnight patients; reoperations occurred in 0% and 8.3%, respectively. Although these contrasts were not statistically significant, the absolute proportions suggest a possible trend toward higher downstream resource use among overnight patients, a hypothesis warranting further evaluation. Likewise, patient-initiated telephone calls or portal messages were recorded in 71.1% of ambulatory patients versus 41.7% of overnight patients. This 29-point difference, while not statistically-significant, may reflect variation in reassurance-seeking behavior and remote communication preferences rather than true differences in postoperative complications. Given that the study was underpowered, failure to achieve statistical significance should not be interpreted as absence of an underlying population effect; instead, these proportions provide preliminary effect-size estimates useful for designing future studies.

An important point that our study adds is the finding of comparable rates of postoperative call or message within 30 days. Since the pandemic, it has been reported clinicians now spend more time after hours working on electronic health records (EHR), which was at least in part due to the 157% increase in messages from patients^29^. This outcome is not unexpected following the COVID-19 pandemic given the push to increase virtual interactions to decrease iatrogenic risk from having patients in clinic and the hospital. While these changes may have decreased unnecessary patient exposure to potential harms from clinical settings, this increased virtual interaction with physicians may cause undue burden on providers without changes to compensation or clinical time. Physicians face a large number of administrative tasks, and it has already been shown that burnout is associated with inbox volume^30,31^. In our study, patients undergoing ambulatory surgery did have a higher rate of reaching out to providers via phone call or message, though this was not significantly different from those who stayed overnight. Given our power and sample size, this pattern should be interpreted as a descriptive observation rather than evidence of equivalence or difference.

For any surgical patient, providing high-quality anticipatory guidance both in the pre- and postoperative setting is critical for reassurance, safety and patient satisfaction, particularly regarding postoperative drain management and incision care. Providing patient-specific discharge instructions with affirming and appropriate gendered terminology is one way to reduce unnecessary health care utilization and support a positive care experience. In our study, absolute rates of post-discharge communication (71.1% in ambulatory vs. 41.7% in overnight) suggest that while earlier discharge did not significantly increase clinician workload, this finding should not be interpreted as evidence of equivalence between groups, given the study’s modest power and unequal group sizes. Rather, these descriptive differences may reflect variable access to telecommunication tools or differing reassurance needs after early discharge.

All patients in this study were discharged under standardized postoperative medication protocols, minimizing variability in pain or antiemetic prescribing. Although the dataset was not structured to capture prescription refills, such events might differ systematically between ambulatory and overnight patients. Within the plastic surgery literature, there is increasing interest in studies of opioid prescribing patterns, particularly in breast and hand surgery populations, exploring associations between ambulatory discharge practices and patient-reported quality-of-care outcomes^32–34^. Although the present study was not structured to analyze this outcome, it highlights an important avenue for future investigation, particularly regarding pain management and post-discharge resource utilization in gender-affirming surgery. In our study, all patients received uniform discharge prescriptions, so the likelihood of systematic prescribing bias may be low; however, undetected differences in refill frequency or real-world consumption could still exist and warrant formal assessment in future work.

An additional finding of note in the present study was a subgroup analysis of patients identifying as nonbinary and their preferences regarding nipple-areola complex (NAC) reconstruction, e.g., free nipple grafts. In total, 20 of 57 patients (35%) in our cohort identified as nonbinary, of whom five (25%) elected to forgo NAC reconstruction. This proportion falls within the range reported in prior studies, which have found that nonbinary patients forgo NAC reconstruction at frequencies ranging from approximately 20% to nearly 50%, depending on institutional context and study design^35–38^. In one such study, Ferrin et al.. observed that 13% of all patients—but nearly half of nonbinary respondents—chose to omit NAC grafts. Other surveys have likewise demonstrated broad variability in preferences along the spectrum of chest procedures. While our study was not powered for formal subgroup inference, the effect size observed (25% vs. 35–50% in prior work) approximates this documented range and highlights the importance of individualized, patient-centered planning. Situating our results within the broader literature underscores the need for collaborative, aesthetic, and gender-expression dialogue within this diverse population.

A salient observation from our study was the frequency of EHR misgendering for TGD patients. With the direct accessibility of clinical notes via electronic portal, such events are now easily visible to patients. Misgendering and/or deadnaming has serious, harmful effects on psychosocial well-being^39–41^. TGD patients, already at higher risk of having poor health care outcomes^42,43^, are less likely to seek out health care when they experience instances of misgendering^44^. Misgendering by health care providers may be attributable to limited experience with this population^45^. As gender-affirming health care providers, it is critical to set an appropriate tone by using accurate name and pronouns in our documentation and verbal handoffs; unfortunately, we cannot be present at every touchpoint in the health care system for our patients.

While the overall frequency of documented misgendering was high, an exploratory subgroup analysis identified a possible difference by discharge status, with inpatient (overnight) patients more often experiencing misgendering than ambulatory patients (66.7% vs. 22.2%). Although this absolute difference of ~ 45% points reached statistical significance, small sample size and potential documentation bias preclude definitive causal inference. The finding suggests a gradient driven by the higher volume of personnel handoffs and templated documentation events in the inpatient setting. Rather than implying absence of risk in ambulatory cases, these results demonstrate that even short-stay care pathways warrant structural safeguards to ensure affirming documentation.

Ongoing interprofessional educational efforts must continue to ensure that all staff members are trained appropriately to work with diverse populations. One way to mitigate this harm is by providing education to hospital staff surrounding this population and the importance of correct use of pronouns^44^. This emphasizes the importance of verbal handoffs with emphasis on gender identity and correct pronoun utilization. At our institution, misgendering or deadnaming most often occurred in notes from members of perioperative nursing or anesthesia teams. This likely indicates an issue with templated notes, which is consistent with well-known criticisms of the use of templates in electronic health records^46^. Therefore, free-texting notes may represent a possible mitigation strategy. The observed effect size between overnight and ambulatory groups underscores how systemic design (such as templating frequency) may influence patient experience independent of clinical outcomes. Our findings highlight the need for structured institutional interventions, including EHR template audits and periodic staff education on affirming documentation practices. Quality-improvement initiatives are being explored to address this issue within our institution.

This study has several limitations. First, this retrospective chart review is prone to human error, which may affect the results. Retrospective designs can only establish associations, not causation. Additionally, because chart review was used, we could not assess certain patient-reported outcomes, an especially important dimension in gender-affirming chest surgery. The study design also precluded structured analysis of post-discharge prescription refills, which may have occurred electronically but were not trackable as discrete data. However, all patients received standardized discharge prescriptions, reducing the likelihood of systematic bias.

The limited cohort size, particularly within the overnight subgroup, yielded low statistical power to detect moderate differences in rare outcomes (e.g., hematoma or wound dehiscence). Thus, nonsignificant p values should not be taken as evidence of no difference but rather as an inability to rule out potentially meaningful population effects. For example, the observed absolute difference of 6.1% points in complication rates, though not statistically significant (*p* = 0.38), could represent a substantial clinical signal if analyzed in larger cohorts. Nonetheless, these findings remain valuable, as research within TGD populations often involves smaller datasets. Our power analysis suggests that while underpowered for rare outcomes, the study captures the full institutional experience during a constrained pandemic period and should be interpreted as exploratory and hypothesis-generating. Finally, this study contains a cohort from one surgeon at a single academic center, which may limit external generalizability.

We acknowledge that the study may be underpowered for broad generalizability due to its limited sample size; nonetheless, these findings remain valuable, as research within the TGD demographic often involves smaller cohorts^47^. Our power analysis suggests that although the study was underpowered to detect moderate differences in uncommon complications, it captures the complete institutional experience during the pandemic period and should be interpreted as exploratory and hypothesis-generating. Finally, this study contains a cohort of patients from one surgeon, which may affect generalizability.

Overall, our study supports ambulatory mastectomy as a potentially safe and feasible alternative to overnight-observation mastectomy in the TGD population. Absolute complication rates were low across both groups (2.2% vs. 8.3%), and while no statistically significant differences were detected, this should not be interpreted as proof of equivalence but rather as an early indicator warranting replication. This transition toward same-day discharge may reduce delays in surgery, lower floor-staff burden, minimize inpatient exposure to iatrogenic harm, and decrease unnecessary health care spending. Importantly, this may be achievable without increased health care utilization or provider burden from post-discharge communication. Within these findings, ambulatory gender-affirming mastectomy emerges as a promising, patient-centered model for post-pandemic surgical practice—but one that still requires confirmatory multicenter trials powered to detect moderate effect sizes and to validate whether observed differences are reproducible at the population level.

The costs of an overnight stay (23-hour observation) are included as part of the procedural payment bundle in some insurance plans. Additional fees incurred are also variable and determined by each hospital system. Our study is intended to provide only safety recommendations for same-day mastectomy, and should be determined case by case, based on each patient’s medical history and social context. As gender-affirming health care providers, we have the privilege of meeting our patients over several visits in the outpatient setting, developing familiarity with their goals and social circumstances. We would advocate that surgeons apply clinical judgment when determining candidacy for same-day discharge. In our practice, this transition has been marked by general success. Future research should integrate these findings with similar studies to develop data-driven decision-support tools for optimizing mastectomy discharge planning.

**Table 1:** Racial demographics.

Patients	Number of patients (*N*)	Total (%)
Total	57	100
White	44	77.2
Black (mean)	7	12.3
Asian/Pacific Islander	3	5.3
Hispanic	0	0
Other/unreported	3	3

**Table 2:** Clinical demographics.

Patients	Ambulatory (*N*, % of all)	Overnight (*N*, % of all)	Total (*N*, % of all)
Total	45 (79)	12 (21)	57
Mean age (years)	26	26	26
BMI (mean)	26.13	25.67	26.03
Smoker	3 (6.7)	0	3 (5.3)
Diabetic	0	1 (8.3)	1 (1.8)

**Table 3:** Perioperative outcomes.

	Ambulatory (*N*, % of ambulatory)	Overnight (*N*, % of overnight)	Total (*N*)				*P*		
Patients	45	12	57						
Surgical complication	1 (2.2)	1 (8.3)	2				0.38		
Hematoma	0	1 (8.3)	1				0.21		
Seroma	0	0	0				–		
Wound dehiscence	1 (2.2)	0	1				1		
SSI	0	0	0				–		
DVT	0	0	0				–		
Return to ED	4 (8.9)	2 (16.7)	6				0.60		
Return to OR	0	1 (8.3)	1				0.22		
Call/message in 30 days	32 (71.1)	5 (41.7)	37				0.42		
Misgendering in EHR	14 (31.1)	8 (66)	22				0.04		

**Table 4:** Factors associated with postoperative complication.

	Patients with complications (*N*, % of subgroup)	*P*
Total patients (*n* = 57)	5 (8.8)	
Incision type		
Periareolar (*n* = 15)	2 (13.3)	0.599
Double Incision (*n* = 37)	3 (8.1)	1
Wise Pattern (*n* = 2)	0	1
Buttonhole (*n* = 3)	0	1
Smoker (*n* = 3)	1 (33.3)	0.245

## Data Availability

The datasets generated during and/or analyzed during the current study are available from the corresponding author on reasonable request.
